# The Feet in Winter

**Published:** 1877-04

**Authors:** 


**THE FEET IN WINTER.**—Sometimes in washing the feet in warm water, a great deal of scurf or whitish soft substance may be scraped from the soles; this is dead skin, dried perspiration, and other accumulations, all resulting from a want of personal cleanliness. These accumulations occur most in winter, when washing the feet is neither as convenient nor agreeable as in summer-time. Many persons suffer from cold feet, simply from a neglect to keep them clean. Few suffer thus in summer-time, one reason for which is that the skin is moist, the pores are open, a free evaporation takes place, and the blood is invited to the surface. In winter the skin is dry, harsh and cold. To keep them constantly warm and comfortable, is indispensable to good health; and to do this, the surface must be brought to the condition of summer; that is, must be soft and somewhat moist, instead of being harsh and dry. This may be soon brought about by soaking the feet in warm water for half an hour at a time daily, using most freely a very stiff brush, with good soap. After the skin has become soft and smooth, a good washing with soap and warm water twice a week during cold weather, will greatly contribute to a healthful condition of the feet as well as to personal comfort. If the feet are kept unexceptionably clean, and are nevertheless inclined to be dry, considerable benefit will be derived by rubbing into the soles every morning a little sweet oil, twenty or thirty drops to each sole, with the palm of the hand, patiently and well, the object being to secure by artificial means, that softness and moistness which is known to favor evaporation and to invite thither the flow of blood. If in addition, the feet were placed in cold water regularly every morning (when not unwell) not over two inches deep, and remaining in not over half a minute in cold weather, then rubbed briskly dry with a coarse cloth, next with the hands, all followed by a brisk walk or stamping for a minute or two, or until they begin to feel comfortably warm after the cold bath, an improvement in the condition of the feet would be secured in a reasonably short time, which would largely compensate for the trouble taken.

